# Histopathologic Evaluation of Periapical Radiolucencies Clinico-Radiographically Diagnosed as Endodontic Lesions: A Retrospective Analysis

**DOI:** 10.30476/dentjods.2023.96819.1967

**Published:** 2024-03-01

**Authors:** Saede Atarbashi-Moghadam, Mehrdad Azar, Shaghayegheh Dowdani

**Affiliations:** 1 Dept. of Oral and Maxillofacial Pathology, School of Dentistry, Shahid Beheshti University of Medical Sciences, Tehran, Iran; 2 Student, Research Committee, School of Dentistry, Shahid Beheshti University of Medical Sciences, Tehran, Iran; 3 Graduated Student, School of Dentistry, Shahid Beheshti University of Medical Sciences, Tehran, Iran

**Keywords:** Odontogenic tumor, Microscopy, Pulpitis, Periapical cyst

## Abstract

**Statement of the Problem:**

Periapical cyst and granuloma are inflammatory endodontic lesions. Periapical granuloma usually heals spontaneously after endodontic treatment; however, periapical cyst mostly needs to be removed via surgical approaches. Although some clinicians believe that microscopic examination of periapical lesions is unnecessary, it is proved that some of them has non-endodontic nature that need critical consideration.

**Purpose:**

The purpose of this study was to assess the disagreement between clinico-radiographic and microscopic diagnosis of periapical cysts and granulomas in a major center of oral pathology service in Iran.

**Materials and Method:**

In this retrospective, descriptive cross-sectional study, the archives of the oral and maxillofacial pathology department of Shahid Beheshti University of Medical Sciences served as the source of the material during an 18-year-period for this retrospective, descriptive cross-sectional study. The reports of all patients whose initial clinical diagnosis was a periapical cyst/granuloma were extracted.

**Results:**

In the present study, 474 cases were diagnosed with a periapical cyst/granuloma clinico-radiographically, of which 61 cases (12.86%) received a microscopic diagnosis of a non-endodontic pathology. The most frequent lesion was odontogenic keratocyst (n= 12, 19.67%) followed by infected odontogenic cyst (n= 12, 19.67%). About 21.31% of diagnoses were non-cystic lesions and 4.9% were malignancies. The most odontogenic tumors that were diagnosed as periapical cyst/granuloma in clinico-radiography were the ameloblastoma variants (n= 4, 6.55%).

**Conclusion:**

A wide variety of microscopic diagnoses, including aggressive lesions such as ameloblastoma, as well as other malignant lesions was noted in this study. These misdiagnoses can lead to an inappropriate treatment plan. It is important to microscopically examine all lesions removed from the jaw.

## Introduction

Periapical lesions are often associated with pulp necrosis leading to inflammatory reaction; however, some of them are developmental cysts and neoplasms [ 1 - 7 ]. Periapical granuloma (PG) as well as common cysts of the jaws including periapical cyst (PC), dentigerous cyst (DC), residual cyst, and odontogenic keratocyst (OKC) have different clinical behaviors. They are caused by inflammatory and developmental pathogenic factors associated with the epithelium of tooth-forming apparatus. PC/PG shows a well-defined unilocular radiolucency encircles the affected tooth apex. Loss of lamina dura and a sclerotic border are also significant radiographic indicators for obtaining a diagnosis. Root resorption can also be observed [ 8 ]. Although tooth vitality is crucial in clinical evaluation, it is important to notice that non-endodontic lesions also may lead to pulp necrosis when located adjacent to root apices. Therefore, the diagnosis cannot be made only on the vitality test of pulp and thorough evaluation of the patient including review of the patient’s past medical and dental history, and accurate assessment of radiographic findings are crucial [ 6 ]. It is recommended to consider cysts and neoplasms in the differential diagnosis of these lesions and to examine all surgically removed periapical lesions microscopically [ 6 - 7 ]. The present study was conducted evaluating the discrepancy between clinic-radiographic and microscopic diagnoses in inflammatory periapical lesions.

## Materials and Method

This retrospective study was approved by the Ethics Committee of Shahid Beheshti University of Medical Sciences (IR.SBMU.DRC.REC.1397.043). The archives of the Oral and Maxillofacial Pathology Department of Shahid Beheshti University of Medical Sciences have been reviewed for 18 years (2001-2018). The inclusion criteria were the files of patients with the initial clinico-radiographic diagnosis of PC/PG. The exclusion criteria were samples without a sufficient demographic data. All slides were extracted and re-evaluated by an oral pathologist (first author) and the previous microscopic diagnosis was confirmed. A descriptive statistical analysis was performed using SPSS version 16 software.

## Results

Within the 18 years, 474 cases had been diagnosed as PC/PGs by clinical and radiographic evidence, of which 61 cases (12.86%) received microscopic diagnosis of a non-endodontic pathology. Overall, there was a slight male predilection with a ratio of 1.17/1 and mandible predominance (ratio: 2.05/1) in the population of non-endodontic periapical lesions. The mean age was 35.39 ranging from 8 to 84 years. The most frequent non-endodontic lesions were OKC (19.67%) and infected (non-specific) odontogenic cyst (19.67%). Glandular odontogenic cysts (GOCs) accounted for 9.83% of cases (Table 1). About 21.31% of diagnoses were non-cystic lesions and 4.9% were malignancies included squamous cell carcinoma (SCC), metastatic adenocarcinoma, and Langerhans cell histiocytosis (LCH). The most odontogenic tumors that were diagnosed as PC/PG in clinico-radiography were the ameloblastoma variants (n=4, 6.55%) (Table 2). 

**Table 1:** Non-endodontic cysts diagnosed as PC/PG in clinic-radiography

Microscopic diagnosis		N (%)	Age range	Gender		Site	
				M	F	Man	Max
Odontogenic cysts	Odontogenic keratocyst	12 (19.67%)	17-60	6	6	8	4
	Dentigerous cyst	10 (16.39%)	8-57	5	5	6	4
	Infected (non-specific) odontogenic cyst	12 (19.67%)	10-62	4	8	8	4
	Glandular odontogenic cyst	6 (9.83%)	8-61	5	1	4	2
	Lateral periodontal cyst	3 (4.91%)	29-40	2	1	3	-
	Calcifying odontogenic cyst	1(1.63%)	28	1	-	-	1
	Buccal bifurcation cyst	1(1.63%)	12	1	-	1	-
Non-odontogenic cysts	Nasopalatine duct cyst	3(4.91%)	35-50	2	1	-	3

**Table 2:** Non-cystic non-endodontic lesions diagnosed as PC/PG in clinic-radiography

Microscopic diagnosis		N (%)	Age range	Gender		Site	
				M	F	Man	Max
Odontogenic tumors	Ameloblastoma (unicystic/solid)	4 (6.55%)	21-54	3	1	4	-
	Granular cell odontogenic tumor	1(1.63%)	57	-	1	1	-
Non-odontogenic lesions	Fibro-osseous lesions	3 (4.91%)	34-42	1	2	3	-
	Central giant cell granuloma	2(3.27%)	20-58	-	2	1	1
	Squamous cell carcinoma	1(1.63%)	84	1	-	-	1
	Langerhans cell histiocytosis	1(1.63%)	50	1	-	1	-
	Metastatic carcinoma	1(1.63%)	64	1	-	1	-

## Discussion

Despite the controversy among dentists regarding the cost and benefit of microscopic examination, the misdiagnosed endodontic periapical lesions consisted of potentially life-threatening lesions, such as ameloblastoma and SCC [ 3 ]. In the present study, the total disagreement between clinico-radiographic and histopathological diagnoses was 12.86%. We reviewed the similar articles and the inconsistency ranged from 0.7% to 19% was reported in previous studies [ 1 , 3 , 9 - 15 ] (Table 3).

**Table 3:** Frequency of non-endodontic periapical lesions (case series) reported in the literature (1990-2021)

Author	Non-endodontic lesion (N, %)	Benign lesions		Malignant tumors
		Odontogenic	Nonodontogenic	
Present study	61 (12.86%)	Keratocyst (12), dentigerous cyst (10), infected odontogenic cyst (12), glandular odontogenic cyst (6), lateral periodontal cyst(3), calcifying odontogenic cyst (1), Buccal bifurcation cyst (1), ameloblastoma (4), granular cell odontogenic tumor(1)	nasopalatine duct cyst (3), fibro-osseous (3), CGCG(2)	Squamous cell carcinoma(1), Langerhans cell histiocytosis (1), metastatic adenocarcinoma(1)
Kosanwat *et al*. 2021 [ 15 ] (Thailand)	157 (10.03%)	Dentigerous cyst (51), keratocyst (31), ameloblastoma (15), Calcifying odontogenic cyst (9), Dental follicle (6), Paradental cyst (3), Orthokeratinized odontogenic cyst (3), Lateral periodontal cyst (2), Glandular odontogenic cyst (2), Adenomatoid odontogenic tumor (1)	nasopalatine duct cyst (18), Foreign body reaction (6), Traumatic bone cyst (4), Fibro-osseous lesions (2), Postoperative maxillary cyst (1)	adenoid cystic carcinoma (1), mucoepidermoid carcinoma (1), metastatic papillary thyroid carcinoma (1)
Guimarães *et al*. 2021 [ 1 ]	208 (19%)	Keratocyst (30), ameloblastoma (24), dentigerous cyst (12), glandular odontogenic cyst (9)	nasopalatine cyst (15), benign fibro-osseous lesion (9)	Carcinoma (5), adenocarcinoma (2), melanoma (1)
Vieira *et al*. 2020 [ 2 ]	306 (4.2%)	Keratocyst (107), dentigerous cyst (48), ameloblastoma (27)	nasopalatine cyst (28), giant cell lesion (22), benign fibro-osseous lesion (16)	Adenoid cystic carcinoma (4), squamous cell carcinoma (3), mucoepidermoid carcinoma (2)
Huang *et al*. 2017 [ 3 ]	118 (3%)	Keratocyst (38), dentigerous cyst (13), ameloblastoma (11), calcifying odontogenic cyst (7)	fibro-osseous lesion (18), nasolabial and nasopalatine cysts (5)	Squamous cell carcinoma (7), adenoid cystic carcinoma (1), Langerhans cell histiocytosis
Sullivan *et al*. 2016 [ 15 ]	166 (2.8%)	Keratocyst, ameloblastoma(2)	fibro-osseous lesion, giant cell lesion (1)	Langerhans cell histiocytosis (1)
Pontes *et al*. 2014 [ 6 ]	11	keratocyst (1), Unicystic am-eloblastoma (1), Myxoma (1)	Simple bone cyst (3), fibro-osseous lesion (2), central giant cell granuloma(1), Nasopalatine duct cyst (1)	Mucoepidermoid carcinoma (1)
Kontogiannis *et al*. 2015 [ 9 ]	52 (3.5%)	Keratocyst (18), glandular o-dontogenic cyst (10), lateral periodontal cyst (6), calcifyi-ng odontogenic cyst (3)	Fibro-osseous lesion (4)	Metastatic carcinoma (1), Langerhans cell histiocytosis (1)
Koivisto *et al*. 2012 [ 7 ]	2587(26.5%)	Keratocyst (857), ameloblastoma (114), lateral periodontal cyst, odontogenic fibroma, calcifying odontogenic cysts, adenomatoid odontogenic tumors	Giant cell lesion (129), neurofibroma, traumatic bone cyst, nasopalatine cyst, Fibro-osseous lesion, and schwannomas	metastasis (25), Muc-o-epidermoid carcinoma, lympho-ma, Langerhans cell histiocytosis, plasm-acytoma, osteosarcoma
Ortega *et al*. 2007 [ 11 ]	26(0.7%)	Keratocyst (11), calcifying odontogenic cyst (1), lateral periodontal cyst (1)	sinusitis (3), giant cell lesion (3), nasopalatine cyst (1), foreign body granuloma (1), cemento-osseous dysplasia (1)	-
Kuc *et al*. 2000 [ 10 ]	8(1%)	Lateral periodontal cyst (1), Pindborg tumor (1), myxoma (1)	Giant cell lesion (2), nasopalatine cyst (1), fibro-osseous lesion (1)	Multiple myeloma (1)
Nobuhara & del Rio 1993 [ 12 ]	(5.7%)	Lateral periodontal cyst	foreign body reaction, sinus tract	-
Spatafore *et al*. 1990 [ 14 ]	66(4%)	keratocyst, odontoma, cementoma,	Actinomycosis, simple bone cyst, nasopalatine duct cyst, ossifying fibroma, central giant cell granuloma, condensing osteitis, chronic osteomyelitis, foreign body reaction	Lymphoma

**Figure 1:**
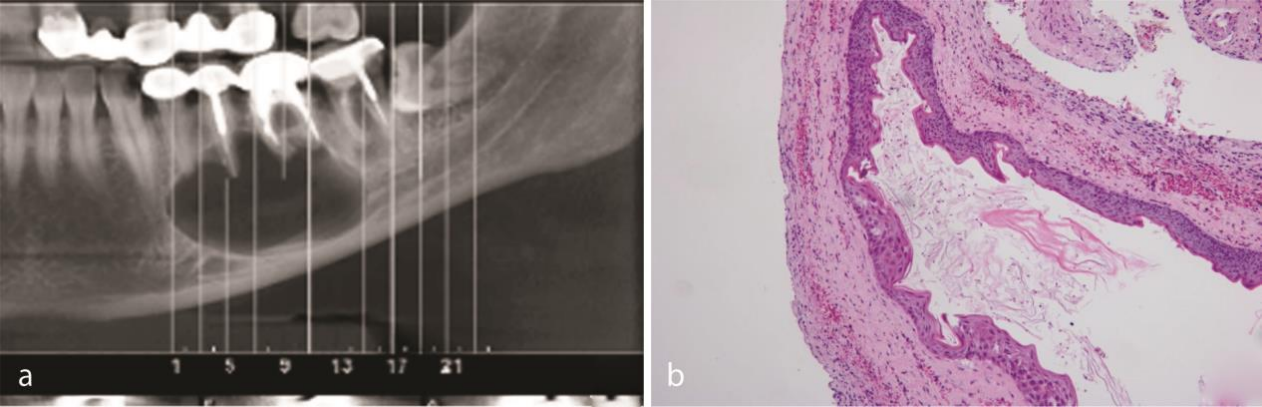
**a:** OKC resembling an endodontic periapical lesion, **b:** Microscopic section shows the characteristic features of OKC (Hematoxylin and eosin, 400×)

**Figure 2:**
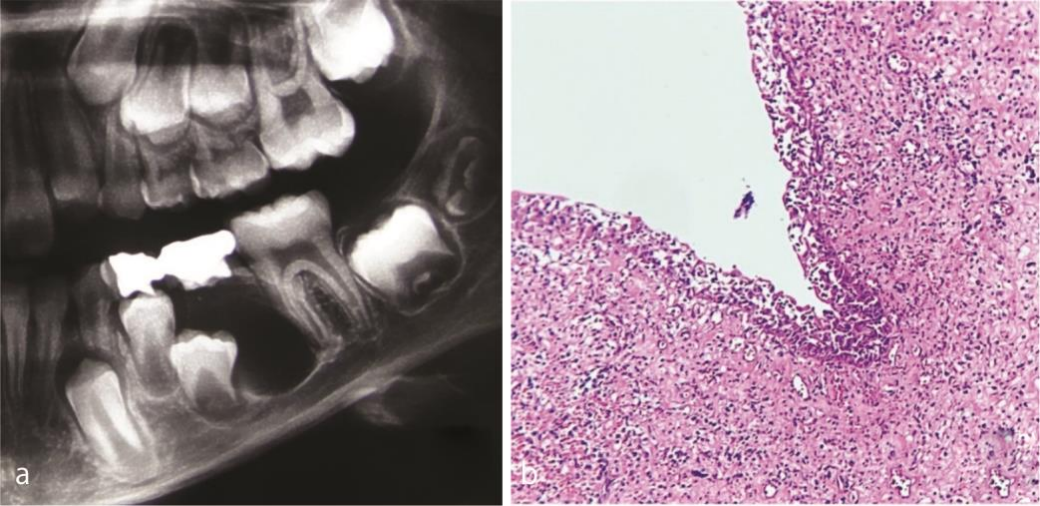
**a:** Dentigerous cyst developed around the crown of an unerupted premolar tooth diagnosed as a periapical cyst of overlying primary tooth, **b:** The microscopic section shows inflamed dentigerous cyst with nonkeratinized stratified squamous epithelium (Hematoxylin and eosin)

In the current study, there was a predilection toward male gender and lower jaw, which was in consistent with Guimarães *et al*. [ 1 ] and Huang *et al*. [ 3 ] studies. However, other reports presented the maxilla as the common location [ 9 , 11 , 15 ]. We found OKC to be most frequently diagnosed as endodontic periapical lesions in clinico-radiological examinations, which was in accordance with previous studies [ 1 - 3 , 9 , 11 , 16 ] (Figure 1). However, DC [ 15 ], simple bone cyst (SBC) [ 6 ], central giant cell granuloma (CGCG) [ 10 ], and lateral periodontal cyst (LPC) [ 12 ] were also reported as the most common misdiagnosed lesions in the other studies. OKC shows a well-defined unilocular or multi-locular radiolucency with a radiopaque rim and scalloped borders. Root resorption of adjacent teeth is infrequent [ 8 ]. These features are not pathognomonic, mostly in smaller unilocular lesions, and may mimic PC [ 17 ]. In the present study, DC accounted for about 16.39% of cases that diagnosed as PC/PG clinic-radiography. DC is typically characterized by a well-circumscribed unilocular radiolucency associated with the crown of an unerupted tooth with a sclerotic border and the most common differential diagnoses include OKC and ameloblastoma. However, DC may occur as a result of periapical inflammation from an overlying primary tooth and may resemble PC of a deciduous tooth; although, PCs that involve the primary tooth are so rare [ 18 ]. Three cases in the current study also showed these features in radiography (Figure 2).

About 9.83% of our cases had a microscopic diagnosis of GOCs, which have been reported 1.27%, 4.32%, and 19.23% in previous studies [ 1 , 9 , 15 ]. GOC typically shows a multilocular radiolucency with a sclerotic rim and scalloped border. Nevertheless, it may demonstrate a unilocular radiolucency, and PC is also considered in its differential diagnosis. The importance of OKC and GOC is that they need more aggressive treatment than PC/PG [ 8 , 15 ]. Aspiration is a useful clinical tool guides the clinician to the better differential diagnosis. Aspiration is highly recommended for moderate to large jaws’ radiolucencies. The cystic lesion shows straw-colored fluid. A pastier content is consistent with keratin and proposes the diagnosis of OKCs or orthokeratinized odontogenic cysts. Blood aspiration suggests a vascular lesion, and a negative aspiration recommends a solid lesion [ 6 ].

**Figure 3:**
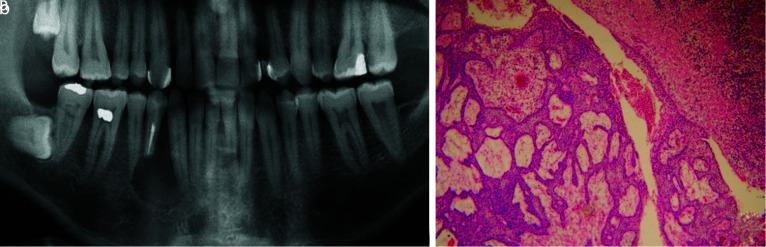
**a:** Unicystic ameloblastoma mimicking an endodontic periapical lesion, **b:** The histopathologic sections show unicystic ameloblastoma (Hematoxylin and eosin, 100×)

Non-cystic lesions comprised 21.31% of the clinico-radiographically diagnosed PC/PG. This finding emphasizes the importance of histopathological examination of periapical lesions. Odontogenic tumors were the mostmisdiagnosed non-cystic lesions (8.19%), and ameloblastoma was the most frequent pathology among them (Figure 3). It is in accordance with Guimarães *et al*. [ 1 ], Huang *et al*. [ 3 ] and Kosanwat *et al*. [ 15 ] findings. Fibro-osseous lesions comprise about 4.91% of the cases, which represented from 1.27% to 15.25% in previous studies [ 1 - 3 , 9 , 15 ]. It is very difficult to differentiate lesions such as focal cement-osseous dysplasia in the early stages from a PC in radiographic evaluation [ 19 ]. In addition, during the lucent phase, the periapical lamina dura is commonly lost [ 6 ]. Vitality test is helpful in the differential diagnosis of these cases [ 19 ].

It is noteworthy that biopsy of cement-osseous dysplasia is not recommended in a classic presentation because of the reduced vascular supply and increased risk of post-operative infection [ 19 ]. CGCG was diagnosed in two cases (3.27%) in the current study, which ranges from 1% to 11.53% in other studies [ 1 , 7 , 11 ]. Dahlkemper *et al*. [ 20 ] mentioned that up to 20% of the CGCGs could be associated with the presence of a tooth with pulp necrosis or a previous endodontic therapy, being a significant differential diagnosis to PC/PG.

In the present study, 4.9% of histopathologic diagnoses were malignant lesions including SCC, metastatic adenocarcinoma, and LCH. The rate of malignancies was different in studies and ranged from 0% to 7% [ 3 , 9 - 12 , 15 ]. SCC [ 3 ], adenoid cystic carcinoma [ 2 ], and metastatic tumors [ 6 ] were mostly reported. Metastatic tumors of the jaws frequently show an ill-defined or moth-eaten radiolucency. Though, they may demonstrate non-aggressive characteristics, representing benign lesions or odontogenic infections. The past medical history may help in detecting the metastatic lesions. However, metastatic tumors in the jaw may be the first sign of primary tumor [ 21 ]. In our study, which is in line with other researches, LCH was diagnosed as a PC [ 3 , 7 , 9 , 16 ]. LCH shows indefinite pathogenesis and a wide range of clinical manifestations and prognoses. Oral LCH clinically may resemble severe periodontitis [ 22 ]. It also can occur inside the jawbone, where they may mimic a periapical inflammatory lesion [ 23 ]. Peters *et al*. [ 23 ] mentioned that LCH should be considered in the differential diagnosis of an apical radiolucency of vital teeth or teeth that do not respond to endodontic therapy. Dentists should be conscious of its clinical and radiographic similarities.

## Conclusion

Although most of the periapical lesions has endodontic origin, this study shows a wide variety of microscopic diagnoses, including aggressive lesions such as ameloblastoma, as well as malignant lesions which was mimicking endodontic periapical lesions clinico-radiographically. This issue emphasizes the precise exploration of the patient's medical and dental histories, using vitality tests of the pulp and aspiration in clinical assessment, and detailed assessment of radiographic findings for achieving a precise diagnosis of periapical lesions. In suspected cases, a biopsy and subsequent microscopic analysis are required. 
